# Effects of Selective Head-and-Neck Cooling on Brain Injury-Related Biomarker Levels and Symptom Rating Following a Boxing Bout: Protocol for an Exploratory Randomized Trial

**DOI:** 10.2196/68954

**Published:** 2025-06-16

**Authors:** Ali Al-Husseini, Yelverton Tegner, Kaj Blennow, Henrik Zetterberg, Niklas Marklund

**Affiliations:** 1 Department of Clinical Sciences Lund Neurosurgery Lund University Lund Sweden; 2 Department of Health, Education and Technology Division of Health and Rehabilitation Luleå University of Technology Luleå Sweden; 3 Department of Psychiatry and Neurochemistry Institute of Neuroscience and Physiology The Sahlgrenska Academy at the University of Gothenburg Mölndal Sweden; 4 Clinical Neurochemistry Laboratory Sahlgrenska University Hospital Mölndal Sweden; 5 Department of Neurodegenerative Disease UCL Queen Square Institute of Neurology London United Kingdom; 6 UK Dementia Research Institute University College London London United Kingdom; 7 Hong Kong Center for Neurodegenerative Diseases Hong Kong China (Hong Kong); 8 Wisconsin Alzheimer’s Disease Research Center University of Wisconsin School of Medicine and Public Health University of Wisconsin–Madison Madison, WI United States; 9 Department of Clinical Sciences Lund Neurosurgery, Lund University Skåne University Hospital Lund Sweden

**Keywords:** sports concussion, biomarker, boxing, glial fibrillary acidic protein, neurofilament-light

## Abstract

**Background:**

Head impacts are common in contact sports such as boxing and occur at times of elevated core body and brain temperatures induced by the exercise. Following impact, elevated brain temperature may lead to the development of exacerbated brain injury that can be monitored by blood biomarkers. Blood-brain biomarkers S100B and glial fibrillary acidic protein (GFAP) reflect glial injury; neurofilament light (NFL), axonal injury; and Neuron-Specific Enolase (NSE) and Tubulin-associated unit (tau), neuronal injury. Time to peak levels post injury for these biomarkers varies. Levels of S100B l peak early post injury, while NSE, GFAP, and tau are regarded as subacute markers, and NFL shows prolonged increases. We attempt to cover a large spectrum of first week postfight alterations in blood-brain biomarkers and their response to head-neck cooling.

**Objective:**

We hypothesized that acute head-and-neck cooling, recently shown to shorten return-to-play in concussed ice hockey players, applied acutely following a boxing bout, is associated with an attenuated concentration of blood biomarkers and improved symptom rating.

**Methods:**

The trial is academically driven and funded by external and hospital research funds. Young, healthy elite boxers aged ≥18 years are recruited. Before, and immediately after a competitive boxing bout consisting of 2 or 3 rounds of 2 minutes each, blood samples are drawn. Boxers are randomized to intervention or control management by 1:1 allocation before baseline testing. After the initial postfight blood sample is drawn and symptom rating using the Sports Concussion Assessment Tool-5 (SCAT-5) has been collected, the boxers receive either acute selective head-and-neck cooling for 45 minutes or routine postfight management. The number of head impacts is counted in all boxers on match video recordings. In both groups, blood samples are drawn 45 minutes after the initial postbout blood sample, as well as 3 and 6 days post fight. At all blood sampling time points, the number of symptoms (NOS) and symptom severity score (SSS) are assessed using the symptom rating part of the SCAT-5. The primary endpoint is the difference in biomarker levels (GFAP, NFL, tau, UCH-L1, neuronal-specific enolase) immediately post fight and preintervention, to those obtained at 6 days post fight. The postfight SCAT-5 NOS and SSS are secondary endpoints.

**Results:**

Recruitment started in November 2021 and is ongoing. So far, 41 boxers have been included: 20 controls and 21 cooled. Data collection started in October 2024 following the completion of blood sample analysis. We expect to recruit more boxers before the middle of 2025, but challenges with recruitment may limit this.

**Conclusions:**

There is no treatment available for boxing-induced brain injury. Biomarkers are surrogate yet objective markers of brain injury, and the head-and-neck cooling treatment may attenuate the concentration of brain injury–related biomarkers as well as reduce symptoms induced by head impacts attained during a boxing fight.

**Trial Registration:**

ClinicalTrials.gov NCT06386484; https://clinicaltrials.gov/study/NCT06386484

**International Registered Report Identifier (IRRID):**

DERR1-10.2196/68954

## Introduction

### Background

Trauma to the head is common in many contact sports, including boxing [1]. If the trauma to the head, or the body with energy transmitted to the head, is sufficient to induce a range of clinical symptoms and signs that may or may not involve loss of consciousness, a sport-related concussion (SRC) has occurred [2]. In boxing, participants both receive and deliver, on average, 32-40 blows to the head during a typical bout [3]. Those blows are referred to as repetitive head impacts (RHI) [4,5]. RHI differs from the definition of SRC by the absence of the acute symptoms typically observed following an SRC. However, similar to after several SRCs cumulative effects of RHI include early-phase changes in brain function and increased risk for neurodegenerative disorders at long-term. Neuropathological consequences of RHIs in boxing have been comprehensively investigated in previous studies, illustrating structural alterations to the brain [2,6]. More specifically, RHI may lead to cognitive impairment, mood disorders, and motor control problems [7-9].

Several studies have shown that retired athletes who have sustained multiple RHI and SRCs during their career may develop chronic traumatic encephalopathy (CTE), a progressive neurodegenerative disease associated with memory loss, depression, personality changes, and dementia [1,8,10-13]. At present, CTE can only be diagnosed post mortem where key findings include irregular aggregation of phosphorylated tau protein at the depths of cortical sulci [8].

In sports, strenuous exercise leads to elevated core body and brain temperature [14], which may exacerbate the brain injury induced by head impacts. Recently, we showed that selective head-and-neck cooling for 45 minutes, aiming to rapidly normalize the elevated brain temperature, applied acutely post SRC in elite ice hockey players, resulted in earlier return-to-play and a smaller proportion of players with prolonged symptoms following the SRC [15]. However, there is a lack of objective outcome measures for assessing the effects of head-and-neck cooling in athletes.

There is a growing body of literature on the use of biomarkers of brain injury in athletes. In Olympic boxers, levels of cerebrospinal fluid biomarkers such as S100 calcium-binding protein (S100B), Tubulin-associated unit (Tau), Neurofilament light (NFL), and Glial fibrillary acid protein (GFAP) were collected at baseline, 1-6 days post fight, and 14 days after RHI attained in a boxing bout [16,17]. In addition, the biomarkers showed both acute and cumulative effects of the impacts, with a lack of normalization of NFL and GFAP after the rest period, which may indicate an ongoing injury process [16,17]. In addition, inflammatory biomarkers such as tumor necrosis factor-alpha, interleukin 6, and muscle injury biomarkers (alanine aminotransferase and aspartate aminotransferase), and creatinine increased in male elite boxers following a fight [18].

When boxing and mixed martial arts (MMA) were compared, retired boxers had higher plasma GFAP levels, and active boxers had higher plasma NFL than MMA fighters [19]. In addition, serum NFL levels were elevated both at 7-10 days post bout and after 3 months of rest in boxers compared to controls. This study also observed a significant increase in NFL at 7-10 days post bout comparing levels obtained after a high number of head impacts (more than 15 hits to the head or experienced grogginess during or after bout) to those obtained after fewer head impacts (fewer than 15 head hits) [20]. These studies indicate that selected biomarkers may remain elevated during the first postinjury week. In this randomized trial, we hypothesized that biomarkers could serve as an objective marker for the efficacy of selective head-and-neck cooling on attenuating brain injury in elite boxers, assessed by blood biomarkers immediately before and during the first 6 days after a competitive boxing fight.

### Objectives

#### Research Hypothesis

The research hypothesis was that acute and selective head-and-neck cooling immediately after a boxing bout attenuated brain injury as evident by a reduced concentration of brain injury-related biomarker levels during the first postfight week.

#### Study Objectives

The primary objective was to determine whether selective head-and-neck cooling attenuates the concentration of brain injury biomarkers.

The secondary objectives were as follows:

1. To determine whether selective head-and-neck cooling improves symptom rating using the Sports Concussion Assessment Tool-5 (SCAT-5).
2. To determine whether any biomarker increase post fight, compared to baseline levels, is associated with the number of head impacts.
3. To determine any adverse event associated with the head-and-neck cooling.

## Methods

### Participants, Interventions, and Outcomes

#### Study Setting

The study will take place predominantly in the southern parts of Sweden due to logistical reasons, aiming for the feasibility of follow-up with biomarkers at distinct postfight time points. Blood samples will be collected at the site of the competitive boxing tournament or the local boxing clubs and transferred to the biobank at Lund University Hospital. All data will be collected in Sweden. The trial is academically driven by local funding. The trial will be conducted at Lund University, Lund, Sweden.

#### Trial Design

The head-and-neck Cooling of Brain temperature In BOXing (COBIBOX) trial is designed as a randomized, controlled, open, single-center superiority trial with 2 parallel groups and a primary endpoint of biomarker changes during the first 6 postfight days. Clinical secondary outcome assessments of a number of symptoms (NOS) and symptom severity score (SSS) using the symptom rating part of the SCAT-5 is performed during the initial 6 postinjury days. Randomization will be performed in blocks with 1:1 allocation.

#### Eligibility Criteria

Eligibility criteria are listed in Textbox 1.

**Inclusion and exclusion criteria.**

**Inclusion criteria**

- Male and female elite boxers <40 years old and ≥18 years old.
- Cleared for participation by the medical staff of the Swedish Boxing Association before the boxing bout.
- Informed consent by each participant before the competitive event.

**Exclusion criteria**

- >40 years old or <18 years old.
- Participation in competitive fights, match-sparring, or any training causing additional head impacts during the 6-day postfight follow-up period after being included in the study.
- History of autoimmune neurological diseases or a neurodegenerative disorder.
- History of previous traumatic brain injury resulting in an intracranial hemorrhage.

#### Procedures and Measurements

**Figure 1:**
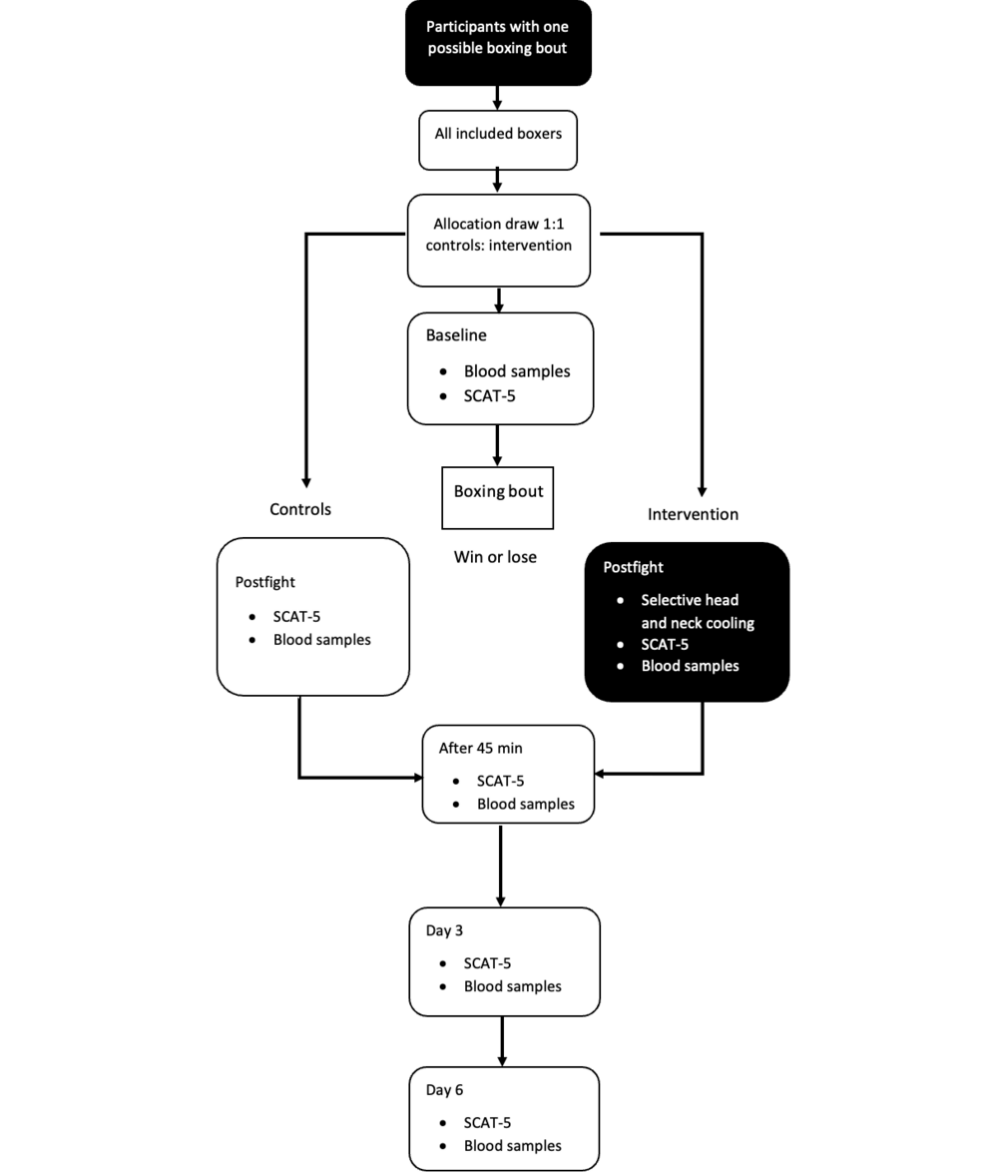
Boxers that participate in direct final bouts or single-fight events will only engage in one boxing bout. SCAT-5: Sports Concussion Assessment Tool-5.

**Figure 2:**
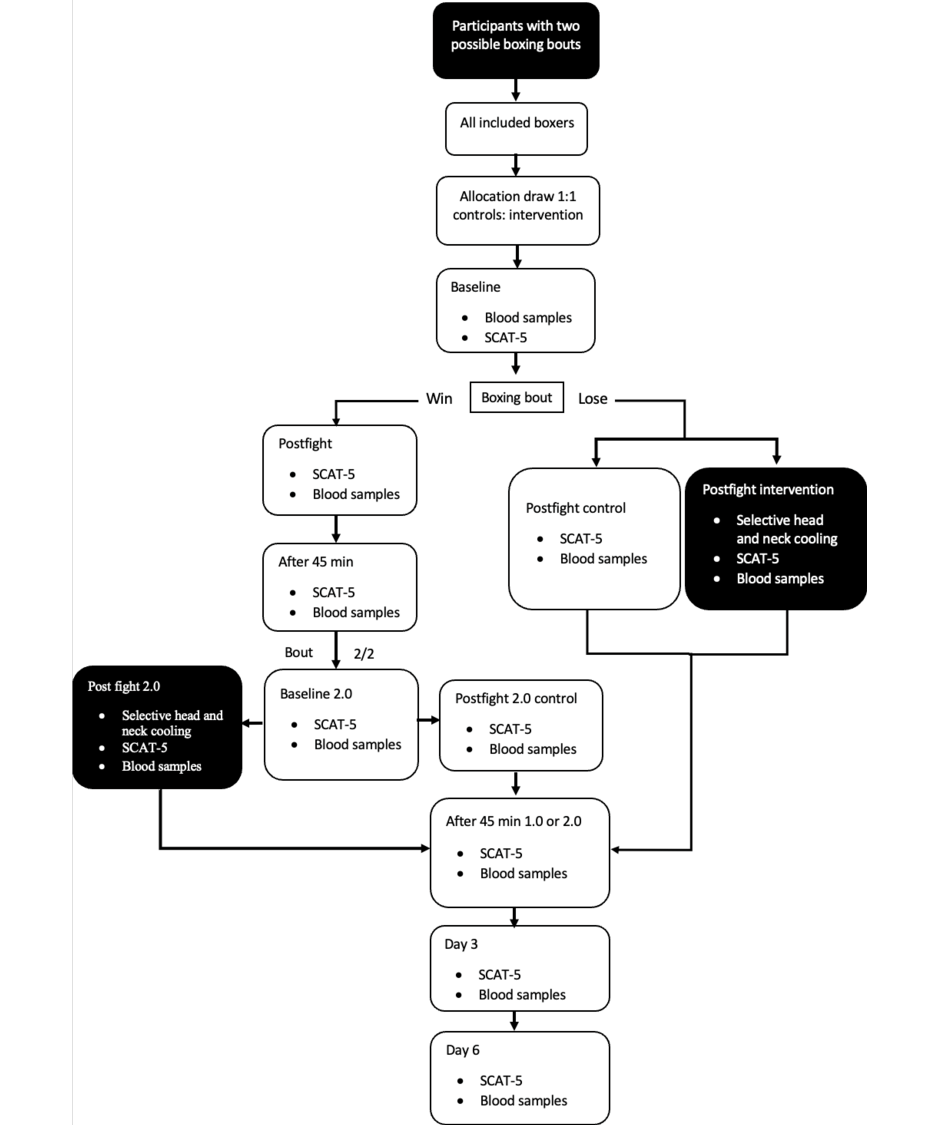
Boxers who participate in tournaments can either fight one or two boxing bouts, depends on whether they win or lose. SCAT-5: Sports Concussion Assessment Tool-5.

All boxing clubs are thoroughly informed of the protocol before study initiation and before each tournament. Boxers between the ages of 18 and 40 years are included. At each tournament, allocation to the treatment takes place on the first morning, and the boxers are randomized 1:1 by a randomly drawn paper slip with a sign that the boxer will receive either selective head-and-neck cooling or routine postfight medical surveillance by an on-site physician. The selective head-and-neck cooling is to be initiated within 10 minutes post fight with a duration of a minimum of 45 minutes. After the boxer completes the fight (Figures 1 and 2), he or she follows the researcher to the cooling station and continues the head-and-neck cooling protocol. In some boxing events, a boxer may participate in two bouts if they win their first bout (Figure 2). The next day, during the finals, they will undergo the same protocol as on their first day, including SCAT-5 assessments and blood sample collection [21]. As mentioned in Figure 2, the start of intervention or return to play protocol starts after their last fight.

Participants will be randomly allocated to either the control or intervention group, involving selective head-and-neck cooling. Baseline SCAT-5 and blood samples will be collected. Subsequently, postfight SCAT-5 and blood samples will be collected for both groups. In addition, the intervention group will undergo selective head-and-neck cooling for 45 minutes. Following this, both the control and intervention groups will follow the same protocol, undergoing SCAT-5 and blood sample collection at 45 minutes post fight, as well as on day 3 and day 6 for follow-up (Figure 1).

The selective head-and-neck cooling takes place after the last boxing bout (dark-colored boxes). For a boxer who fights bout 2/2, new baseline SCAT 5 and blood samples will be collected, shown as baseline 2.0 in Figure 2.

**Figure 3:**
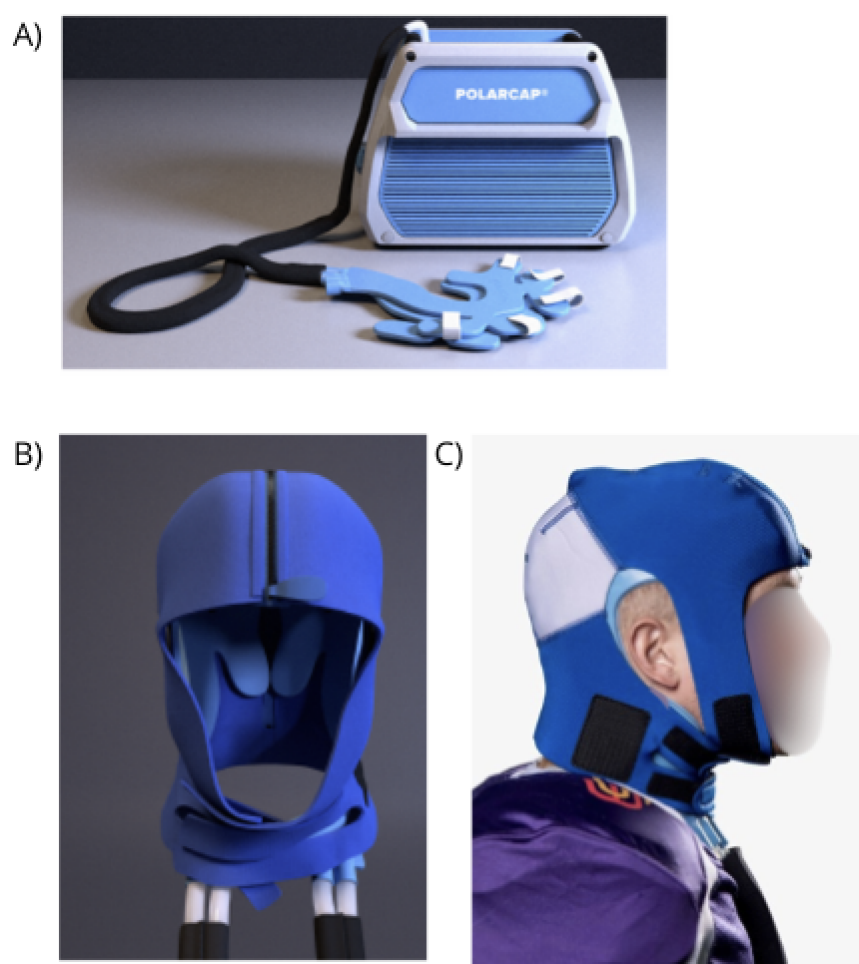
(A) The PolarCap unit is a powerful mobile cooling system. (B) The Polar Coolant is transported through the silicone head cap. (C) To insulate against the cold, a neoprene cover is applied on top of the cap.

The PolarCap System consists of a portable cooling system, designed to reduce brain temperature by controlled cooling of the scalp and neck with a circulating coolant (PolarCap Coolant, PolarCool AB). The coolant is maintained at 0 °C and flows through a silicone-based head cap. An insulating neoprene cover is put on top of the cap for isolation. The players are allowed to relax (sitting or supine) as long as the cooling head cap and neoprene cover remain in place on the head [22] Should the boxer experience discomfort and wish to discontinue the intervention, the cap is removed and the athlete is excluded from the study. All boxers follow the return-to-fight protocol. The standardized protocol is to return to physical exercise during the first 6 postfight days. During this time, the boxer may do all forms of physical exercise except sparring, match fight, or other activities resulting in impacts to the head or body (Figure 3).

Blood samples are collected at the following time points for all participants: the morning of the first boxing fight on day 1 (baseline, at rest), immediately after the fight, 45 minutes after the fight, and after 3 and 6 days in all boxers. The blood samples obtained at 3 and 6 days post fight will be drawn by a study nurse or a researcher (AaH) at the participants’ boxing club. The samples are transported to the hospital in a mobile refrigerator to maintain a temperature of 5-8 °C. Thereafter the serum samples are centrifuged at 3000 rpm, at 4 °C for 10 minutes, after which 800-1200 µL is transferred using a pipet to 2-mL tubes and stored at –80 °C.

At each time point for blood sample collection, boxers are also administered the SCAT-5 questionnaire, which is a self-report questionnaire with information on 22 different symptoms, each with a severity grading of 0-6, where 0 indicates no symptoms and 5-6 indicates severe symptoms. The maximum total score is 132. All boxing bouts are video-recorded for later analysis. The total hits per bout, strikes to the head, strikes to the body, and visible evidence of neurological disturbance (loss of consciousness, impaired balance or coordination, vacant expression, etc) will be assessed. After completion of the study, the boxers will be interviewed by phone to answer questions about their previous boxing, knock-out, and concussion history. The timeline for enrollment intervention and assessments across the study is visualized in Table 1 (SPIRIT [Standard Protocol Items: Recommendations for Interventional Trials] checklist in Multimedia Appendix 1).

**Table 1:** Timeline of enrollment, intervention, and assessment during the study period.

Procedures		Study period				Post allocation						Close-out
		Enrollment	Preallocation	Allocation								
Time point		–t_1_	Baseline	0	Post fight		45 min post fight	Day 3	Day 6	1 week post fight		
**Enrollment**		✓										
	Eligibility screen	✓										
	Informed consent	✓										
	Basic medical examination	✓										
	Allocation			✓								
**Interventions**												
	Selective head and neck cooling (A^a^)				✓							
	Return to fight protocol (A+B^b^)				✓		✓	✓	✓			
	Collecting blood sample		✓		✓		✓	✓	✓			
**Assessments (SCAT-5^c^)**												
	SCAT-5		✓									
	SCAT-5 follow-up				✓		✓	✓	✓			
	Boxing history									✓		

^a^A: intervention group.

^b^B: control group.

^c^SCAT-5: Sport Concussion Assessment Tool-5.

### Ethical Considerations

The study outlined in this article will be carried out in accordance with the Declaration of Helsinki. Regional ethics committee approval in Lund, Sweden (decision number Dnr 2022/06195) was obtained. Before their enrollment in the trial, all study participants will provide written informed consent. Each participant signs the form, containing comprehensive study details. Approval from the Swedish Boxing Association to include amateur and professional boxers registered in the Swedish Boxing Association (SBA) in the study will be received before the study is initiated. Forms describing the study are sent to all boxing clubs in southern Sweden that would participate in the tournaments and have enrolled boxers over 18 years old. All participants are informed of their right to terminate their participation in the study at any given time without the need to provide a reason for doing so. All questionnaires and data collection methods were reviewed and approved by the Swedish Ethical Review Authority in Uppsala, Sweden. The submitted protocol to the Swedish Ethical Review Authority included detailed information on the intervention, data confidentiality, blood collection, blood sample storage, and measures to ensure the protection of participants’ privacy. A study protocol was provided with the ethical application. All participants' data are anonymized from the time of inclusion, before allocation, by assigning a code containing letters and numbers. This code will be attached to the participants’ blood samples and SCAT-5 score files. Each participant will receive a compensation of 1000 SEK (US $104.53) for their time, inconvenience, and any potential discomfort associated with participation. The consent information emphasizes the voluntary nature of participation, including the possibility of opting out at any stage. It also specifies who will obtain informed consent from the participants. Specific contact information for the research team is provided to participants for any questions or concerns. The informed consent consists of 10 detailed points. It begins with a request for participation that describes what the boxers will be involved in. The background and aim section explains the purpose of the study and the facts supporting it. The study procedure outlines the collection of SCAT-5 questionnaires, blood samples, and the possibility of cooling. Biomarker collection is covered by the biobank policies and includes the category of biomarkers that will be analyzed. The risk section highlights any potential risks associated with participation. Handling personal data explains data protection measures in accordance with GDPR, while data management and privacy further detail how data are stored and covered under GDPR. The insurance section clarifies the responsible parts that ensure the participants during the study. Voluntary participation ensures that participants are informed of their right to opt out at any time, even after inclusion. Finally, the responsible staff section provides information about the staff in charge and their contact details.

### Study Population

A total of 14 boxing clubs whose fighters enrolled in 5-7 boxing events in southern Sweden will be contacted and informed about the study and its inclusion criteria.

### Allocation

After all boxers have approved participation in the study during the tournament, and signed the informed consent, the study staff makes an allocation on paper slips that equals the number of participants. Then the boxers randomly, by drawing a paper slip, receive either a sign for receiving head-and-neck cooling (C=cooling) or NC (no cooling; Figures 1-2). All allocations are finalized a minimum of 3 hours before the start of the tournaments.

The enrollment of participants will be done by a researcher (AaH) aided by boxing coaches.

After the boxing bout, and after the boxer is evaluated for red flags mandating transport to the nearest hospital, the boxer is brought to the intervention station to apply the PolarCap system as previously described.

### Personnel Responsible for Informed Consent

Informed consent documents will be sent for approval to the Swedish Boxing Association, after which those will be distributed to each club. A responsible researcher and physician (AaH) will inform the participants verbally and in written form, either days before the competition or on the same day. Each participant will be informed about the study, informed of the possibility to withdraw from participation at any time, and provided time to ask questions. Before randomization, each participant signs the informed consent form.

### Biomarker Analysis

Venous blood is collected in 3.5 mL serum and EDTA plasma tubes generated at room temperature and stored in a mobile refrigerator at 4-8 ºC until centrifuged at 4 ºC for 10 minutes at 3000 rpm. The remaining supernatant is transferred to 2 mL cryotubes and stored at –80 ^o^C until analyzed. Once the study has been completed, blood biomarkers for brain injury (S100B, GFAP, tau forms, NFL, and NSE) will be measured in coded samples using an established single-molecule array (for GFAP, tau, and NFL) by Quanterix and electrochemiluminescence immunoassays (for S100B and NSE) by Roche Diagnostics immunoassays [23] in the Clinical Neurochemistry Laboratory by board-certified laboratory technicians who will be blinded to the intervention.

### Data Collection

The SCAT-5 form will be filled out at the time of biomarker analysis and used as a secondary endpoint of the study. The boxing bouts take place either in events consisting of single bouts for each boxer or in tournaments where 2 bouts are possible (semifinal and final). For boxers participating in two bouts at the same event, the baseline, postfight, and 45-minute postfight scores and biomarker tests will be retaken. Values related to the second fight will be considered in the analysis. The analysis of symptom severity scores obtained by SCAT-5 at the set timeline will facilitate the interpretation of outcomes between the boxers independent of their previous boxing history after the completed 6-day follow-up. Boxing history will be obtained by phone call interview at the 6-day follow-up or on-site. The follow-up for days 3 and 6 will be organized by the local clubs before the first postmatch training session, or at the study hospital.

### Sample Size and Statistical Power

Based on previous reports, an approximately 10% increase in key biomarkers is observed after a concussion in athletes [16]. We hypothesized a 20% reduction of biomarker level by acute head-and-neck cooling. We assumed equal variance of the cooled and control groups. The selected power was set to 0.8. Power analysis was conducted using the following formula:



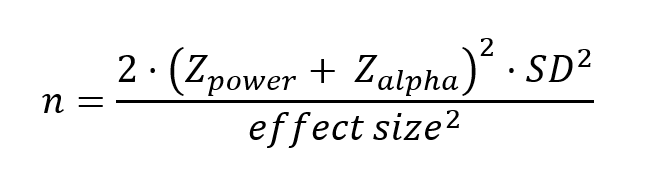



[24]

Based on the power calculations, a total number of 20 participants per group is expected to yield sufficient statistical power. Since dropouts and missed sample times can be anticipated we aim for group sizes of 25 per group.

### Recruitment

Participants are recruited by one researcher (AaH) by sending information about the study to the Swedish Boxing Association which approves participation, in written form or verbally. Thereafter the study must be approved by the organizers of the boxing tournament. Finally, the recruitment of boxers who fulfill the inclusion criteria and sign the written consent form after verbal and written information about the study is provided.

### Adverse Events

Any adverse events reported by the participant during the study period are noted and reported. Specifically, any deviation from the protocol (eg, sample time point, duration of head-and-neck cooling) will be noted. Symptoms, such as headache, nausea, and dizziness, are reported to the medical doctor and study researcher (AaH), although these symptoms may also be caused by the SRC per se [15]. No adverse events are expected, based on our previous study where 61 elite ice hockey players were cooled without any reported adverse events [15].

## Results

**Figure 4:**
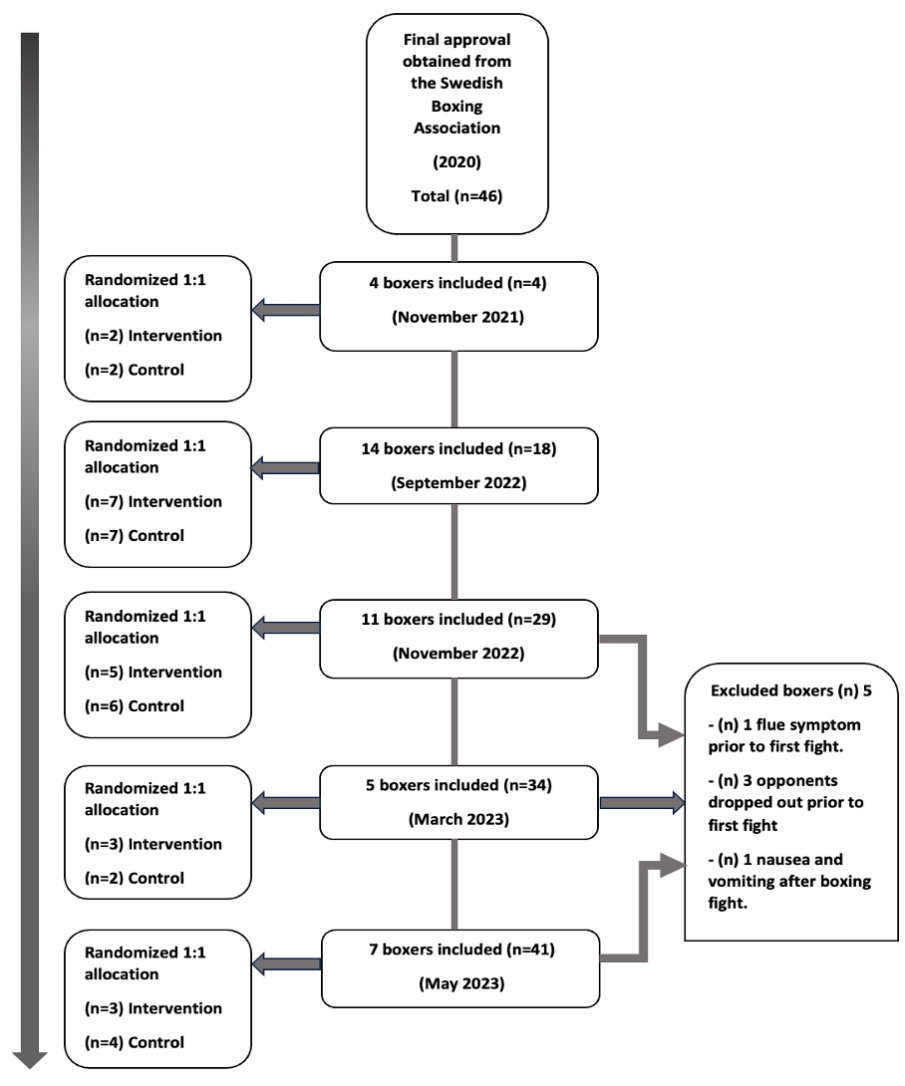
All allocation and randomization occur after signed informed consent and before baseline testing.

The study flow is presented in Figures 1 and 2. The study began in November 2021 and is expected to be completed before the summer of 2025 (Figure 4). Based on our sample size calculations, we aim to recruit approximately 40-50 participants. The first results are anticipated to be submitted for publication in the Autumn of 2025. This study evaluates the effectiveness of selective head and neck cooling in reducing blood-brain biomarkers and SCAT-5 scores during the first week after a boxing bout. Recruitment started on November 4, 2021. Approximate completion of final recruitment is expected in the first weeks of January 2025 (protocol version 1.0, 2024-04-01).

The 3 excluded boxers, whose opponents dropped out, went through baseline SCAT-5 testing and blood sample collection. The participant who experienced nausea and vomiting chose to withdraw from participation in the study after his boxing bout before any further testing (Figure 4).

## Discussion

### Principal Findings

During a boxing fight, boxers typically attain a high number of blows to the head, which may lead to brain injuries. Due to the strenuous activity, these blows occur at a time of elevated core body temperature, which may exacerbate the brain injury. There are no approved treatments for boxing-induced brain injuries. A blow to the head may lead to an elevation of blood biomarker concentrations that reflect the degree of the brain injury caused by the impact. There is an increasing interest in these biomarkers that include markers for large caliber axon injury (NFL), neuronal injury (NSE, tau), and glial injury (S100B, GFAP). Our present study may show whether the level of biomarkers is related to the number of hits or concussions received, the symptom development after the fight, and most importantly the effects of the cooling intervention.

Head-and-neck cooling was shown to decrease brain temperature measured by magnetic resonance imaging (MRI) [25], and improved cognitive outcome when applied acutely post concussion [26]. In a recent clinical trial including 132 concussed ice hockey players, a shorter recovery time was observed after acute selective head-and-neck cooling for at least 45 minutes [15]. A shorter recovery was also observed when cooling was initiated up to 8 days post concussion [27]. To provide an objective outcome measure for the cooling intervention in the present study, the primary outcome is blood biomarker levels, of which GFAP, NFL, S100B, and tau may be the most important. The chosen time points for collecting blood-brain biomarkers aim to assess the early-phase biomarker response to boxing matches, with subsequent evaluation of the head and neck cooling effect and its potential early impact. S100B is associated with an acute astrocytic response, which typically peaks within hours, followed by GFAP with a peak of 24 hours [28]. This makes postfight day 3 a valuable time point to capture a delayed peak or sustained elevation of the selected biomarkers. While NFL levels may remain elevated at longer time points post fight, we had to use 6 days as our latest evaluation time point. This is based on the guidelines of the Swedish Boxing Association, which prohibit boxers from competing or having fights within 6 days of their most recent tournament. Thus, many boxers are assigned to match sparring or other tournaments after 6 days.

The selection of biomarkers in the present trial is based on several previous studies [29,30], mainly in traumatic brain injury and neurodegenerative disorders [19]. Currently, both tau isoforms and GFAP are used in the prediction and diagnosis of neurodegenerative diseases, particularly Alzheimer disease, and S100B in mild traumatic brain injury guidelines [31-33].

In numerous reports on mild, moderate, or severe traumatic brain injury, blood and CSF biomarkers have been used at different time points postinjury [29]. Following boxing bouts, tau, NFL, GFAP, and S100B are all increased in the early phase after boxing. Of these, NFL, GAP, and tau are increased several weeks to months post fight [16,17,34].

In addition, strenuous exercise has been reported to increase biomarkers such as S100B [35,36]), while leading to a reduction in GFAP and no alteration in NF-L levels [37]. When comparing strenuous exercise to contact sports such as boxing, boxers showed a significant increase in biomarker levels, particularly in GFAP and NFL [17].

Blood S100B levels were significantly elevated in both competitive boxers and high-level exercised athletes such as runners [16,38]. These findings argue that boxing causes the elevation of concentration of several biomarkers reflecting the brain injury, and is associated with lead to long-term neurological consequences.

The role of brain temperature in acute brain injury has been evaluated in numerous trials. In particular, elevated brain temperature may negatively impact functional and neurological outcomes following traumatic brain injury [39,40]. While systemic hypothermia has not been associated with improved outcomes in severe traumatic brain injury, we hypothesized that controlling body temperature may be an important factor in the recovery of boxing-induced brain injury. The primary aim of head-and-neck cooling is not to provide cerebral hypothermia, it is to attenuate exercise-induced hyperthermia and to rapidly normalize the elevated brain temperature.

During exercise, elevated temperature is associated with a higher demand for oxygen and energy metabolism in the brain [41,42], resulting in exacerbation of the injury mechanism initiated by head impacts during boxing. We hypothesize that brain cooling can rapidly normalize brain temperature, thereby reducing metabolic demand resulting in attenuated secondary injury and leading to beneficial effects for the recovery phase after a fight.

Should this explorative study provide evidence of the beneficial effects of head-neck cooling, a multicenter study (preferably with later postfight time points) is warranted.

### Strengths and Limitations

The present study has the potential to objectively evaluate the effect of head-and-neck cooling on blood biomarker levels. Moreover, it can provide unique results on biomarker concentration changes following repeated head impacts at several time points post fight.

Outliers in biomarker concentrations are expected, although since each boxer will be his or her control, percentage changes from baseline can be calculated. A 20% reduction of biomarker concentrations is reasonable based on the rapid application of head-and-neck cooling. The potential placebo effect of cooling on symptom ratings is also taken into consideration by using objective biomarker concentrations, highlighting the necessity of biomarker assessment to evaluate cooling’s physiological impact.

### Conclusion

This study is structured to evaluate early-phase objective and subjective changes in boxers during the first week post fight, and to study the effects of head-and-neck cooling on these changes. In conclusion, the present trial may provide objective biomarker evidence for the role of attenuating cerebral hyperthermia by selective head-and-neck cooling acutely following a boxing bout.

## Data Availability

The datasets generated or analyzed during this study are available from the corresponding author on reasonable request.

## Appendix

Multimedia Appendix 1 SPIRIT (Standard Protocol Items: Recommendations for Interventional Trials) checklist COBIBOX.

## Abbreviations

COBIBOX: Cooling of Brain temperature In BOXing
CTE: chronic traumatic encephalopathy
GFAP: glial fibrillary acid protein
MRI: magnetic resonance imaging
MMA: mixed martial arts
NSE: neuron specific enolase
NFL: Neurofilament light
NOS: number of symptoms
RHI: repetitive head impacts
S100B: S100 calcium-binding protein
SCAT-5: Sports Concussion Assessment Tool-5
SRC: sport-related concussion
SBA: Swedish Boxing Association
SPIRIT: Standard Protocol Items: Recommendations for Interventional Trials
SSS: symptom severity score
Tau: tubulin-associated unit
