# Supplementary material for: Effects of Selective Head-and-Neck Cooling on Brain Injury-Related Biomarker Levels and Symptom Rating Following a Boxing Bout: Protocol for an Exploratory Randomized Trial
Source: JMIR Res Protoc. 2025 Jun 16;14:e68954. doi: 10.2196/68954 (PMC12209727; doi:10.2196/68954)
Supplement: Multimedia Appendix 1 [file resprot_v14i1e68954_app1.pdf]

STANDARD PROTOCOL ITEMS: RECOMMENDATIONS FOR INTERVENTIONAL TRIALS

SPIRIT 2013 Checklist: Recommended items to address in a clinical trial protocol and related documents*

| Section/item | Item No | Description |
|---|---|---|
| **Administrative information** | | |
| Title | 1 | **Selective head-and-neck cooling on biomarker levels and symptom rating following a boxing bout- protocol for an exploratory randomized trial.** |
| Trial registration | 2a | Study protocol registered at www.ClinicalTrials.gov, NCT06386484.<br><br>Registered in 2019.<br><br>Protocol version 1 from April 2, 2024 |
| | 2b | All items from the World Health Organization Trial Registration Data Set… please see pages. 1, 5 and 8. |
| Protocol version | 3 | Research protocol version 1.0, dated 2 April 2024 |
| Funding | 4 | This study is academically sponsored by grants to the first and senior author from ALF-LUA, Lund University, Skane University Hospital, Sweden, Sweden. Please see page 14. |
| Roles and responsibilities | 5a | Names, affiliations, and roles of protocol contributors… please see pages 1 and 14. |
| | 5b | Niklas Marklund, Senior Consultant, professor at Department of Clinical Sciences Lund, Neurosurgery<br>Skåne University Hospital EA-blocket plan 3<br>Entrégatan 7, 222 42 Lund, Sweden<br>niklas.marklund@med.lu.se, +46 725 95 02 61 |

| | | |
|---|---|---|
| | 5c | Role of study sponsor and funders, if any, in study design; collection, management, analysis, and interpretation of data; writing of the report; and the decision to submit the report for publication, including whether they will have ultimate authority over any of these activities … Please see page 9, 12 and 15 |
| | 5d | Composition, roles, and responsibilities of the coordinating centre, steering committee, endpoint adjudication committee, data management team, and other individuals or groups overseeing the trial, if applicable (see Item 21a for data monitoring committee)… please see page 9, 11, 12 and 15 |
| **Introduction** | | |
| Background and rationale | 6a | Description of research question and justification for undertaking the trial, including summary of relevant studies (published and unpublished) examining benefits and harms for each intervention …please see pages 2, 3, 4 |
| | 6b | Explanation for choice of comparators…. Please see pages 2, 3,4 |
| Objectives | 7 | Specific objectives or hypotheses… Please see pages 5 and page |
| Trial design | 8 | Description of trial design including type of trial (eg, parallel group, crossover, factorial, single group), allocation ratio, and framework (eg, superiority, equivalence, noninferiority, exploratory)…please see page 5 and figures 1-2 |
| **Methods: Participants, interventions, and outcomes** | | |
| Study setting | 9 | Description of study settings (eg, community clinic, academic hospital) and list of countries where data will be collected. Reference to where list of study sites can be obtained… please see pages 5-6 |
| Eligibility criteria | 10 | Inclusion and exclusion criteria for participants. If applicable, eligibility criteria for study centres and individuals who will perform the interventions (eg, surgeons, psychotherapists)…please see pages 6. |
| Interventions | 11a | Interventions for each group with sufficient detail to allow replication, including how and when they will be administered… please see pages 7-8. |
| | 11b | Criteria for discontinuing or modifying allocated interventions for a given trial participant (eg, drug dose change in response to harms, participant request, or improving/worsening disease) Please see pages 6-7. |
| | 11c | Strategies to improve adherence to intervention protocols, and any procedures for monitoring adherence (eg, drug tablet return, laboratory tests)…please see page 9 |

| | | |
|---|---|---|
| | 11d | Relevant concomitant care and interventions that are permitted or prohibited during the trial… please see page 6. |
| Outcomes | 12 | Primary, secondary, and other outcomes, including the specific measurement variable (eg, systolic blood pressure), analysis metric (eg, change from baseline, final value, time to event), method of aggregation (eg, median, proportion), and time point for each outcome. Explanation of the clinical relevance of chosen efficacy and harm outcomes is strongly recommended… please see pages 5 |
| Participant timeline | 13 | Time schedule of enrolment, interventions (including any run-ins and washouts), assessments, and visits for participants. A schematic diagram is highly recommended … please see figures 1-2. |
| Sample size | 14 | Estimated number of participants needed to achieve study objectives and how it was determined, including clinical and statistical assumptions supporting any sample size calculations… please see page 10. |
| Recruitment | 15 | Strategies for achieving adequate participant enrolment to reach target sample size… please see pages 12. |

**Methods: Assignment of interventions (for controlled trials)**

Allocation:

| | | |
|---|---|---|
| Sequence generation | 16a | Method of generating the allocation sequence (eg, computer-generated random numbers), and list of any factors for stratification. To reduce predictability of a random sequence, details of any planned restriction (eg, blocking) should be provided in a separate document that is unavailable to those who enrol participants or assign interventions… please see pages 8-9 and figures 1-2. |
| Allocation concealment mechanism | 16b | Mechanism of implementing the allocation sequence (eg, central telephone; sequentially numbered, opaque, sealed envelopes), describing any steps to conceal the sequence until interventions are assigned… please see page 9. |
| Implementatio n | 16c | Who will generate the allocation sequence, who will enrol participants, and who will assign participants to interventions… please see pages 7,9. |
| Blinding (masking) | 17a | Who will be blinded after assignment to interventions (eg, trial participants, care providers, outcome assessors, data analysts), and how… please see pages 9 and 10. |
| | 17b | If blinded, circumstances under which unblinding is permissible, and procedure for revealing a participant's allocated intervention during the trial… please see page 7, 9. |

**Methods: Data collection, management, and analysis**

| | | |
|---|---|---|
| Data collection methods | 18a | Plans for assessment and collection of outcome, baseline, and other trial data, including any related processes to promote data quality (eg, duplicate measurements, training of assessors) and a description of study instruments (eg, questionnaires, laboratory tests) along with their reliability and validity, if known. Reference to where data collection forms can be found, if not in the protocol… please see pages 4-5 |
| | 18b | Plans to promote participant retention and complete follow-up, including list of any outcome data to be collected for participants who discontinue or deviate from intervention protocols… please see pages 4-5 |
| Data management | 19 | Plans for data entry, coding, security, and storage, including any related processes to promote data quality (eg, double data entry; range checks for data values). Reference to where details of data management procedures can be found, if not in the protocol… please see page 10 and 11. |
| Statistical methods | 20a | Statistical methods for analysing primary and secondary outcomes. Reference to where other details of the statistical analysis plan can be found, if not in the protocol… please see page 11. |
| | 20b | Methods for any additional analyses (eg, subgroup and adjusted analyses)… no subgroup analyses are planned. |
| | 20c | Definition of analysis population relating to protocol non-adherence (eg, as randomised analysis), and any statistical methods to handle missing data (eg, multiple imputation)… not applicable. |
| **Methods: Monitoring** | | |
| Data monitoring | 21a | Composition of data monitoring committee (DMC); summary of its role and reporting structure; statement of whether it is independent from the sponsor and competing interests; and reference to where further details about its charter can be found, if not in the protocol. Alternatively, an explanation of why a DMC is not needed… please see pages 15 and 16. |
| | 21b | Description of any interim analyses and stopping guidelines, including who will have access to these interim results and make the final decision to terminate the trial… no interim analyses are planned. |
| Harms | 22 | Plans for collecting, assessing, reporting, and managing solicited and spontaneously reported adverse events and other unintended effects of trial interventions or trial conduct… please see page 12. |
| Auditing | 23 | Frequency and procedures for auditing trial conduct, if any, and whether the process will be independent from investigators and the sponsor… No auditing planned. |

**Ethics and dissemination**

| | | |
|---|---|---|
| Research ethics approval | 24 | Plans for seeking research ethics committee/institutional review board (REC/IRB) approval… please see page 8 and translated ethics file. |
| Protocol amendments | 25 | Plans for communicating important protocol modifications (eg, changes to eligibility criteria, outcomes, analyses) to relevant parties (eg, investigators, REC/IRBs, trial participants, trial registries, journals, regulators) … no modifications are planned. |
| Consent or assent | 26a | Who will obtain informed consent or assent from potential trial participants or authorised surrogates, and how (see Item 32)… please see page 9. |
| | 26b | Additional consent provisions for collection and use of participant data and biological specimens in ancillary studies, if applicable… not applicable. The original consent form covers all data that will be collected during the course of the trial, including blood-sampling that may or may not be used for additional ad-hoc analyses. No additional endpoints requiring consent are planned. |
| Confidentiality | 27 | How personal information about potential and enrolled participants will be collected, shared, and maintained in order to protect confidentiality before, during, and after the trial. Specified in the ethical application. There is a strict adherence to GDPR protocols. |
| Declaration of interests | 28 | Financial and other competing interests for principal investigators for the overall trial and each study site… please see pages 15, 16 and 17. |
| Access to data | 29 | Statement of who will have access to the final trial dataset, and disclosure of contractual agreements that limit such access for investigators… please see page 15. |
| Ancillary and post-trial care | 30 | Provisions, if any, for ancillary and post-trial care, and for compensation to those who suffer harm from trial participation… please see pages 2 and 9. |
| Dissemination policy | 31a | Plans for investigators and sponsor to communicate trial results to participants, healthcare professionals, the public, and other relevant groups (eg, via publication, reporting in results databases, or other data sharing arrangements), including any publication restrictions… please see page 6 |
| | 31b | Authorship eligibility guidelines and any intended use of professional writers… please see page 6 |
| | 31c | Plans, if any, for granting public access to the full protocol, participant-level dataset, and statistical code… please see page 6 |

**Appendices**

| | | |
|---|---|---|
| Informed consent materials | 32 | Model consent form and other related documentation given to participants and authorised surrogates… please see page 3 and page 5 in additional file 1 |
| Biological specimens | 33 | Plans for collection, laboratory evaluation, and storage of biological specimens for genetic or molecular analysis in the current trial and for future use in ancillary studies, if applicable…. All analyses fall under the primary endpoint of biomarkers. No genetic analyses are planned. |

*It is strongly recommended that this checklist be read in conjunction with the SPIRIT 2013 Explanation & Elaboration for important clarification on the items. Amendments to the protocol should be tracked and dated. The SPIRIT checklist is copyrighted by the SPIRIT Group under the Creative Commons "Attribution-NonCommercial-NoDerivs 3.0 Unported" license.
